# Inhibiting Microbial Toxins Using Plant-Derived Compounds and Plant Extracts

**DOI:** 10.3390/medicines2030186

**Published:** 2015-07-31

**Authors:** Abhinav Upadhyay, Shankumar Mooyottu, Hsinbai Yin, Meera Surendran Nair, Varunkumar Bhattaram, Kumar Venkitanarayanan

**Affiliations:** Department of Animal Science, University of Connecticut, Storrs, CT 06269, USA; E-Mails: abhinav.vet@gmail.com (A.U.); Shankumar.mooyottu@uconn.edu (S.M.); Hsin-bai.yin@uconn.edu (H.Y.); meera.nair@uconn.edu (M.S.N.); varunkumar.bhattaram@uconn.edu (V.B.)

**Keywords:** plant-derived compounds, plant extracts, bacterial toxins, mycotoxins, toxin inhibition, anti-virulence strategies

## Abstract

Many pathogenic bacteria and fungi produce potentially lethal toxins that cause cytotoxicity or impaired cellular function either at the site of colonization or other locations in the body through receptor-mediated interactions. Various factors, including biotic and abiotic environments, competing microbes, and chemical cues affect toxin expression in these pathogens. Recent work suggests that several natural compounds can modulate toxin production in pathogenic microbes. However, studies explaining the mechanistic basis for their effect are scanty. This review discusses the potential of various plant-derived compounds for reducing toxin production in foodborne and other microbes. In addition, studies highlighting their anti-toxigenic mechanism(s) are discussed.

## 1. Introduction

Microbial infections are a leading cause of morbidity and mortality in humans worldwide. Every year, microbial infections cause more than 100 million illnesses worldwide [1]. Antibiotics have been traditionally used for treating microbial illnesses. However, their excessive and inappropriate use has led to the development of antibiotic resistance in microbes, thereby making the treatment of infections a formidable challenge [2,3]. Moreover, antibiotics such as fluoroquinolones are contraindicated in certain clinical conditions such as hemolytic uremic syndrome since their use leads to induction of Shiga toxin-encoding bacteriophages, increased expression of Shiga toxin genes and toxin release [4,5]. Therefore, research efforts to search for alternative therapies to control microbial infections in humans have intensified.

Microbial illnesses are orchestrated by an array of virulence factors that contribute to the pathophysiology and survival of the pathogen in the host [6]. These factors include cell surface proteins, receptors, and adhesins that facilitate host colonization; capsular polysaccharides that confer protection from host immune system, and microbial toxins that cause host tissue damage [7,8]. Reducing the expression of these virulence factors could control infections in humans. With increasing understanding of microbial virulence factors, pathogenesis, and cell-to-cell communication, interference with anti-virulence strategies is a compelling approach as it circumvents the development of resistance by applying less selective pressure on microbes as compared to traditional approaches, which are aimed at killing bacteria [9]. Since microbial toxins play a prime role in the virulence and pathogenesis of microbes in the host, they are major targets for developing such therapeutic interventions.

Plant-derived compounds represent an untapped source of safe, effective, and environmentally friendly antimicrobials. The properties of various plant-derived antimicrobials that target cellular viability of microbes have been adequately discussed previously [10,11,12,13], but very few reviews have highlighted the potential of plant-derived antimicrobials as effective anti-virulence strategy agents for reducing toxin production in microbes. In this review, we have focused on the effects of plant-derived antimicrobials on microbial toxins, both bacterial and fungal, critical for pathogenesis in the host. In addition, studies highlighting the anti-toxigenic mechanism(s) of plant compounds are presented.

## 2. Microbial Toxins: Diversity, Structure and General Mechanisms of Action

Microbial pathogens such as bacteria and fungi mediate host pathogenesis either through their invasive properties or toxigenic nature or both. A number of pathogenic microorganisms produce toxigenic substances that alter normal cellular metabolism of the host leading to various disease processes [14,15]. Toxins (endotoxins and exotoxins) produced by bacteria or mycotoxins synthesized by pathogenic fungi act as major virulence factors of a variety of microbial pathogens [16,17].

Why microbes produce toxins is still an interesting question. The diversity among the microbial toxins suggests that complex evolutionary pathways are involved in the emergence of toxigenic microorganisms. Moreover, a majority of the bacterial toxins exert no known physiological role in bacterial cells other than damaging host cell [6,16]. A popular school of thought is that bacterial toxin genes evolved from primitive eukaryotic functional genes that were incorporated to bacterial genome, and hence have similar functions or substrates. Another hypothesis is that bacteria evolved to produce toxigenic proteins that can target specific eukaryotic cells in order to acquire selective advantage over their host for niche or survival [16,18]. In addition, the evolutionary process through horizontal transfer of virulence/toxin genes enabled different bacterial groups to modify their genomes resulting in emergence of pathogenic strains [19,20]. Bacterial toxins are broadly classified as exotoxins and endotoxins based on their site of action/release.

### 2.1. Exotoxins

Biochemically, bacterial exotoxins are often proteins, mostly having an **A**–**B** structural and functional organization [14]. These toxins are released from the bacterial cell which enable them to interact with the site of infection or tissues distant from the focus of infection, subsequently injuring the host by a variety of mechanisms [14].

Generally, the A-subunit of the exotoxins has catalytic activity similar to enzymes, and hence is responsible for the toxigenic property of the toxin. The specific catalytic activity of the A-subunit defines the specific tissue or organ target, based upon which the bacterial toxins are classified as enterotoxins, neurotoxins, and leukotoxins. While several bacterial toxins, including tetanus and botulinum neurotoxin, are strictly tissue specific in their activity, many of the *Clostridial* toxins are broad-spectrum with less tissue specificity and potent cytotoxicity in the host cell [21,22,23]. The A-subunit of toxins such as hemolysins and leukocidins has broad spectrum phospholipase activity responsible for its non-specific membrane targeted activities [24,25]. B-subunit is responsible for toxin binding and transfer of enzymatically active A-subunit to the interior of the host cell. Both subunits are usually linked by a disulfide bond or non-covalent interactions [14].

One of the major groups of bacterial exotoxins is pore forming toxins. These toxins are produced by pathogens such as *Listeria monocytogenes* (Listeriolysin O), *Escherichia coli* (hemolysins), *Staphylococcus* (alpha toxin), and other bacteria producing cholesterol-dependent cytolysins (CDC) [14,26]. Pore formation in the plasma membranes of host cells such as erythrocytes or enterocytes causes influx and/or efflux of small molecules resulting in osmotic imbalance leading to cellular swelling, disruption and cell death, which often result in secondary inflammatory reaction in the tissues [15,26]. Another class of bacterial exotoxins is the heat stable enterotoxins (ST) that are produced by pathogenic strains of microbes such as *E. coli*, *Klebsiella*, and *Vibrio*. These toxins bind to the guanylate cyclase C (GC-C) receptor on the surface of the enterocytes, and activates protein kinases to initiate a downstream signaling cascade resulting in increased chloride ions in the extracellular space which cause fluid accumulation and diarrhea [14,27]. Toxins produced by pathogenic *Shigella* and *E. coli* (Stx 1 & Stx 2) exert their toxigenic activity by inhibiting protein synthesis [15]. *N*-glycosidase activity of the A-subunit Stx cleave 28S ribosomal rRNA and arrests protein synthesis [28]. Similarly, diphtheria toxin inhibits protein synthesis by ADP-ribosylation of elongation factor-2 [29].

A variety of bacterial toxins also act through the second messenger pathways in host cells, thereby eliciting injury in the host cell. For example, *Clostridial* toxins such as *C. difficile* toxins, and cytotoxic necrotizing factors from *E. coli* modify cellular Rho family GTPase leading to structural and functional disruption of the host cells and tissues [21,30]. Certain *Clostridial* toxins such as botulinum toxins and tetanus toxins exert their effects by inhibiting the release of neurotransmitters [21]. In addition, a group of bacterial exotoxins known as superantigens produced by bacteria such as *Staphylococcus aureus* bind major histocompatibility complex II (MHC II) of the antigen presenting cells and cause immune activation leading to toxic shock [31].

### 2.2. Endotoxins

Another major group of bacterial toxins are endotoxins. Bacterial endotoxins are lipopolysaccharides (LPS) present in the outer membrane of Gram-negative bacteria such as *E. coli*, *Salmonella*, *Shigella*, and *Pseudomonas*. These toxins are liberated following the lysis of the bacterial cell [32]. LPS consists of a glycolipid domain known as Lipid A, an oligosaccharide core and outer O-antigen. Lipid A domain is recognized by Toll-like receptor 4 (TLR-4) of host cells which in turn triggers downstream signaling pathways culminating in the synthesis and release of proinflammatory cytokines such as TNF-α and IFN-β. These molecules cause widespread vascular and inflammatory changes in the host cell, leading to endotoxin shock [32]. Endotoxin shock is a serious medical condition resulting from sepsis caused by bacterial infections [33].

Most bacterial toxin synthesis genes and other genes responsible for the regulation of toxin gene expression are encoded/located in distinct regions of the bacterial DNA called pathogenicity loci or pathogenicity islands [6,19,34]. Some bacterial toxins are also encoded by extrachromosomal plasmids, bacteriophages and other mobile genetic elements. As mentioned before, toxin genes in the pathogenicity loci of one bacterium can be transferred to other bacteria by horizontal gene transfer, thereby sharing/transmitting virulence factors among strains [6,19].

## 3. Plant-Derived Antimicrobials

Since ancient times, plants have played a key role in the development of human civilization. Besides being used as food preservatives, flavor enhancers and dietary supplements, plant extracts have been used in traditional medicine for treating various metabolic and infectious diseases. Fossil records date the medicinal use of plants at least to the middle Paleolithic age around 60,000 years ago [35,36]. Today, plant extracts are extensively used for preparing drugs (e.g., digoxin, morphine, vincristine), bioactive compounds (metformin, nabilone), pharmacological tools (yohinbine, lysergic acid diethylamide) and herbal remedies (cranberry extracts, garlic) for various medicinal applications [36]. However, this usage accounts for only 6% of the total estimated number (approximately 250,000) of higher plant species on earth [37]. Thus, plants represent a vast, untapped natural resource of useful chemicals.

The antimicrobial activity of several plant-derived compounds has been previously reported [10,38,39,40], and a wide array of active components has been identified [41]. A majority of plant-derived antimicrobials are secondary metabolites, and are produced in response to chemical stimulus from pathogenic microbes or environmental cues [42]. Being secondary metabolites, these compounds do not directly modulate plant physiology [43], however, they play a critical role in enhancing plant health and defense [44]. The generation of secondary metabolites with medicinal properties is restricted to a limited set of species and developmental period within a phylogenetic group as compared to primary metabolites, which are more commonly produced [45].

Although the exact pathways by which plant compounds exert their antimicrobial effects are not clearly defined; multiple, dose-dependent mechanisms have been reported. At bactericidal concentrations, plant compounds cause membrane damage, loss of energy production, enzyme dysfunction and leakage of cellular contents that lead to impaired cellular physiology and cell death [46]. In addition, recent research has revealed that at sub-lethal or sub-inhibitory concentrations, plant compounds affect virulence in Gram-positive [47,48,49] and Gram-negative bacteria [50] and fungal pathogens [51] by modulating gene transcription [52,53], protein expression [53] and quorum sensing [54,55]. Thus, plant compounds represent a viable strategy to develop anti-virulence agents that target toxin production.

## 4. Studies Highlighting the Anti-Toxin Properties of Plant-Derived Antimicrobials

Plant compounds have been found to be effective in reducing toxin production in both bacterial and fungal pathogens. The major studies which have investigated the antitoxin potential of plant compounds are discussed below, and are summarized in Table 1.

### 4.1. Effect of Plant Compounds on Gram-Positive Bacterial Toxins

#### 4.1.1. *Clostridium difficile*

*Clostridium difficile* is a nosocomial pathogen that causes a severe, toxin-mediated colitis in humans, especially in patients undergoing prolonged antibiotic therapy [56,57]. In the United States, approximately 300,000 cases of *C. difficile-*associated disease are reported annually costing more than $3 billion in healthcare costs [58]. The hypervirulent, highly toxigenic *C. difficile* strain known as ribotype 027 is responsible for causing a majority of *C. difficil*e infections reported in the United States [59]. Long-term antibiotic therapy results in the disruption of the normal gut microflora, leading to the germination of *C. difficile* spores and colonization of vegetative cells in colon to cause severe toxin-mediated colitis [59]. *Clostridium difficile* toxins, TcdA and TcdB, with the help of TcdE, a holin-like protein needed for toxin excretion, causes disruption of the cytoskeleton and colonic epithelial tight junction subsequently resulting in severe inflammation, epithelial necrosis, pseudomembrane formation and diarrhea [60,61]. The sigma factor TcdR positively regulates toxin production, whereas TcdC is an antagonist of TcdR, and thus helps to control toxin production [30,62,63]. In addition, CodY, a global pleiotropic repressor, reduces toxin production in *C. difficile* [64,65]. Not many studies have investigated the effect of plant compounds on toxin production in *C. difficile*. In a recent study, carvacrol (obtained from oregano oil) and *trans*-cinnamaldehyde (major component of cinnamon) were found to reduce *C. difficile* toxin production and toxin-mediated cell cytotoxicity without affecting the growth of normal gut flora *in vitro* [49]. Follow up gene expression studies revealed that these compounds down-regulated toxin production genes. In addition, the compounds were found to modulate transcription of toxin repressors favorably. Furthermore, mutant analysis revealed that the plant compounds reduce toxin production potentially through global repressor CodY [49]. Since toxin is the only major virulence factor in *C. difficile*, the antitoxigenic properties of carvacrol, *trans*-cinnamaldehyde or similar plant compounds on *C. difficile* deserve further scientific attention.

**Table 1:** Studies illustrating the efficacy of plant-derived compounds and plant extracts in reducing toxin production in microbes with potential mechanism(s) of action.

Microbe	Toxin(s)	Plant Compounds with Anti-Toxin Activity	Potential Mechanism of Action/Target Site	Reference(s)
Gram positive bacteria				
*Clostridium sp.*	TcdA, TcdB	Carvacrol, *trans*-cinnamaldehyde,	Down-regulation of toxin production genes, modulation of transcriptional repressor	[49]
	Botulinum neurotoxin	Toosendanin		[66,67]
*Bacillus sp.*	Labile enterotoxin	Carvacrol,	Modification of bacterial membranes.	[68,69,70,71]
	Anthrax lethal toxin	Celastrol, toosendanin	Inhibition of toxin entry to cell cytoplasm	[72,73]
*Staphylococcus aureus*	Hemolysin Enterotoxin A, B Toxic shock syndrome toxin	Essential oils from clove, cinnamon, oregano, *Zataria multiflora*, eugenol, 4-hydroxytyrosol	Reduced expression of toxin production genes, *sea*, *seb*, *tst*, *hla*	[53,74,75,76,77]
*Listeria monocytogenes*	Listeriolysin O (LLO)	*trans*-Cinnamaldehyde, carvacrol, thymol, eugenol, oil of bay, clove, nutmeg, thyme	Down-regulation of *hly* and *prfA* genes coding for toxin production and transcriptional regulator	[47,78,79]
*Vibrio cholerae*	Cholera toxin	Tea catechins, Dihydroisosteviol	Modulation of transmembrane regulators	[80,81]
		RG-tannin, apple phenols	Inhibition of ADP-ribosyltransferase activity	[82,83]
		Red chilli, sweet fennel, white pepper Red bayberry, thymol, carvacrol, eugenol	Modulation of toxin production genes *ctxA*, *tcpA*, *toxT*	[84,85,86,87]
Toxin producing *E. coli*	ETEC toxin	Extracts from *Galla Chinensis* and *Berberis aristata*, leanolic acid, ursolic acid, and betulinic acid, essential oil from *Cymbopogon martini*, *C. winterianus* and *Psidium guajava*	Inhibiting intestinal secretion of ETEC enterotoxins Blocking the binding of heat labile enterotoxin to GM1 Reducing toxin binding and toxin mediated cellular pathology	[88,89,90,91,92,93,94,95,96,97]
	Verotoxin	Extracts from *Limonium californicum (Boiss.) A. Heller*, *Cupressus lustianica Miller*, *Salvia urica Epling* and *Jusiaea peruviana L.*, eugenol, catechin, epigallocatechin, cinnamon bark oil, cinnamaldehyde, *Curtisia dentata* extract *carvacrol*, *thymol*, *beta-resorcylic acid*, grape seed and pomace extracts	Decrease in toxin production Reducing the transcription of *stx1* and *stx2* genes Reducing the expression of globotriaosylceramide (Gb3) receptor by mimicking toxin receptors	[98,99,100,101,102,103,104,105,106,107,108,109]
*Aspergillus ochraceus* *Penicillium verrucosum*	Ochratoxin A	Oregano, mint, basil, sage, and coriander	Inhibiting fungal growth	[110]
*Penicillium expansum*	Patulin	Garlic, thyme, lavender oils, *Azadirachta indica* extracts.	Inhibiting fungal growth, mycelium formation and sporulation	[111,112]
*Fusarium graminearum*	Zearalenone	Cinnamon, clove, oregano, palmarosa, and lemongrass oils	An aromatic nucleus and phenolic OH group of plant compounds disrupting fungal cell membrane	[113]
	Deoxynivalenol	Clove		[113]
*Fusarium proliferatum*	Fumonisin B	Extracts from maize, garlic, and pea	Inhibiting primary and secondary metabolism of the fungal pathogens, decreasing biomass formation	[114]
Aspergillus flavus Aspergillus parasiticus	Aflatoxins	Clove, cinnamon, *Zataria multiflora*, carvacrol, *trans*-cinnamaldehyde	Down-regulation of aflatoxin production genes including *aflc*, *ver1*, *nor1* and *norA* genes	[51,115,116,117]

#### 4.1.2. *Bacillus sp.*

*Bacillus sp.* is a significant public health concern due to their ability to form resistant endospores and toxins. Over the last 30 years, these organisms have undergone massive taxonomic improvements and now over 56 genera and over 500 species of *Bacillus* are identified [118,119]. Among the disease-causing *Bacillus*, *B. cereus* is well known as a cause of foodborne infections and intoxications. Although *B. licheniformis*, *B. subtilis* and *B. pumilus* have also been isolated from food associated illnesses, their role in infections still remains uncertain [119]. A few strains of *B. cereus* are reported to cause foodborne illness known as “fried rice syndrome” in humans. *B. cereus* foodborne intoxications are commonly contracted from fried rice stored at room temperature for several hours [119,120]. Foodborne illness caused by *B. cereus* normally manifests as diarrheal and/or emetic type. The diarrheal type is caused by a labile enterotoxin and the emetic type is caused by heat stable emetic toxin [118,119].

Ultee and coworkers studied the effect of carvacrol on *B. cereus* growth, cellular physiology and toxin production *in vitro* [68,69,70] as well as its ability to reduce toxin production in food matrices, including rice and soup [70,71]. These researchers observed that carvacrol at a concentration below its MIC was able to reduce *B. cereus* toxin production *in vitro* [70]. Follow up studies in food matrices showed that the effect of carvacrol in reducing toxin production persisted in soup and rice [69,70]. In addition to its effect on toxin production, carvacrol was found to modulate the fluidity and lipid composition in the membranes of the pathogen, which could be responsible for reduced survival in food matrices [69].

In addition to inhibiting *B. cereus* toxin production, plant compounds have also shown efficacy against *B. anthracis* toxins. For example, Chapelsky and coworkers [72] reported that celastrol, a quinone methide triterpene derived from *Tripterygium wilfordii* Hook F. (Chinese thunder of God vine), inhibited anthrax lethal toxin-induced cytolysis of murine macrophages RAW264.7. Similarly, Slater and coworkers [73] identified novel compounds that protect from anthrax lethal toxin-induced cell death. These researchers observed that Toosendanin (triterpenoid from *Melia azedarach*) was effective in inhibiting the entry of anthrax lethal toxin into the cell cytoplasm without affecting cellular physiology. Toosendanin was also shown to possess *in vivo* efficacy against botulinum neurotoxin [66,67].

#### 4.1.3. *Staphylococcus aureus*

*Staphylococcus aureus* produces a wide range of exotoxins that facilitate its pathogenesis in the host. These include hemolysins (alpha, beta, gamma, and delta), nucleases, proteases and collagenase that mediate host tissue digestion for bacterial nourishment and growth. In addition, *S. aureus* produces enterotoxins, exfoliative toxins and toxin shock syndrome toxin-1 that exert stress on host immune system and also on vital organs [121]. Controlling the production of many of these toxins either in food products or in the host would significantly contribute to reducing *S. aureus* virulence and pathogenesis.

As observed with *Clostridium* and *Bacillus sp.*, plant compounds at their sub-inhibitory concentrations were found to modulate toxin production in *S. aureus*. Essential oils from clove and cinnamon reduced *S. aureus* alpha-hemolysin, enterotoxin A and enterotoxin B production *in vitro* [74]. Qiu and coworkers [53] reported similar findings and observed that eugenol (active component in clove oil) significantly reduced the production of alpha hemolysin, enterotoxin A and B, and toxic shock syndrome toxin *in vitro.* In addition, eugenol was found to reduce the expression of toxin producing genes, including *sea*, *seb*, *tst*, *and hla* coding for various stages of toxin production in *S. aureus.* Similar antitoxin effect has been observed with low concentrations of other plant compounds, including *Origanum vulgare* or oregano [75], *Zataria multiflora* Boiss, a spice plant found in mid-west Asia [76] and 4-hydroxytyrosol [77].

#### 4.1.4. *Listeria monocytogenes*

The foodborne pathogen *Listeria monocytogenes* produces a non-enzymatic, cytolytic, thiol-activated, pore-forming toxin called Listeriolysin O. The toxin is most active at an acidic pH of 5.5 [122] that is usually found inside a phagolysosome. The toxin ruptures the phagolysosome and helps in the escape and survival of the pathogen in the cytoplasm. Listeriolysin O is encoded by the *hly* gene and is activated by the master transcriptional regulatory protein PrfA. Several studies have investigated the efficacy of plant-derived antimicrobials on Listeriolysin production in *L. monocytogenes.* Filgueiras and Vanetti [78] investigated the effect of sub-lethal concentrations of eugenol on Listeriolysin production in *L. monocytogenes.* Using sheep red blood cells based titration assay, these researchers observed a dose-dependent reduction in Listeriolysin production with eugenol. Similar results were obtained with sub-lethal concentrations of oil of *bay*, *clove*, *cinnamon*, *nutmeg* and *thyme* [79]. Follow up gene expression analysis revealed that plant compounds such as *trans*-cinnamaldehyde, carvacrol, and thymol (obtained from oregano oil) at sub-inhibitory or sub-lethal concentrations modulated the expression of *hly* and *prfA* genes that code for the toxin and master regulator, respectively, without affecting growth of the pathogen [47].

### 4.2. Effects of Plant-Derived Antimicrobials on Toxins Produced by Gram-Negative Microbes

As compared to Gram-positive bacteria, relatively a larger number of studies has investigated the efficacy of plant compounds in reducing toxin production in Gram-negatives, especially *Vibrio* spp. and *E. coli* O157:H7.

#### 4.2.1. *Vibrio cholerae*

*Vibrio cholerae* is a water-borne pathogen that is the causative agent of human cholera. It is a significant problem in countries where the sanitary conditions are poor. Manifestation of cholera in humans is through watery diarrhea accompanied by abdominal cramps and severe dehydration. The virulence in cholera is primarily mediated by an AB_5_ protein toxin called cholera toxin [123,124]. After ingestion, the pathogen binds to intestinal epithelial cells using toxin co-regulated pilus (tcpA). Once attached, the pathogen secretes cholera toxin that is internalized by intestinal epithelial cells, followed by processing in the endoplasmic reticulum. Dissociation of A and B subunit leads to ADP-ribosylation of the alpha subunits of the heteromeric G_s_ proteins, resulting in the activation of the adenylate cyclase pathway [125,126,127]. This causes an increase in chloride ion secretion and blocking of sodium influx resulting in an increased solute concentration in the lumen leading to severe diarrhea.

In the past, plant extracts and/or their active components have been used to reduce toxin production in *Vibrio* spp. Toda and coworkers [80] investigated the effect of tea catechins on toxin production by *V. cholerae.* These researchers observed that the plant compounds significantly reduced fluid accumulation induced by cholera toxin in adult mice and intestinal loops of rabbits. Similar results were observed by Pariwat and coworkers [81] who found that dihydroisosteviol reduced cholera toxin mediated intestinal fluid secretion *in vitro*. Apical chloride current measurement indicated that dihydroisosteviol reversibly targeted the cystic fibrosis transmembrane regulator. Follow up *in vivo* studies using a mouse closed-loop model showed that intraluminal injection of dihydroisosteviol significantly reduced intestinal fluid secretion without altering cell viability or fluid absorption. Other plant compounds such as RG-tannin and apple phenols have also shown efficacy in reducing toxin induced fluid accumulation in mouse ileal loop assay [82,83]. Mechanistic studies revealed that the RG-tannin and apple phenols inhibited ADP-ribosyltransferase activity, which is critical for cholera toxin action.

In recent years, several researchers have investigated the effect of plant-derived compounds on the transcriptional profile of *V. cholerae* to elucidate their mechanism in inhibiting cholera toxin production. Yamasaki and coworkers [84] investigated the effect of spices such as red chilli, sweet fennel, and white pepper on cholera toxin production. All the spices significantly modulated cholera toxin production. Capsaicin was found to be the major component among the tested spices and significantly reduced the expression of critical virulence genes including *ctxA*, *tcpA*, and *toxT* that code for toxin production in *V. cholerae.* Interestingly, Capsaicin also enhanced the expression of *hns* gene [85] that negatively regulates toxin production genes, indicating that the effect of plant compounds in reducing toxin production in *V. cholerae* could be due to their effect on the whole transcriptome of the pathogen. A similar efficacy in modulating the expression of genes critical for toxin production has been observed with other plant compounds such as red bayberry [86], thymol, carvacrol, and eugenol [87].

#### 4.2.2. Toxin Producing *Escherichia coli*

*Escherichia coli* are a large and diverse group of bacteria that are found in environment, foods and intestine of humans and animals. A majority of *E. coli* live as harmless commensals, however, some are pathogenic due to the presence of virulence factors acquired over evolution. The major pathogenic groups include enterotoxigenic (ETEC), enteropathogenic (EPEC), enterohemorrhagic (EHEC), enteroinvasive (EIEC), diffusely adherent (DAEC) and enteroaggregative *E. coli* (EAEC) [128]. These cause potentially fatal illnesses in humans characterized by gastroenteritis, septicemia, and meningitis [129]. *E. coli* O157:H7 (EHEC) is the most important serotype among the different pathogenic groups, and is responsible for approximately 73,000 foodborne illnesses annually in the US [130]. The pathogenesis of EHEC and other virulent *E. coli* except for enteroinvasive *E. coli* is chiefly mediated by the production of toxins [129,131]. In the case of EHEC, verotoxins are the major virulence factors that cause intestinal and renal epithelial damage leading to hemorrhagic colitis and hemolytic uremic syndrome, respectively [132,133]. Enteroaggregative *E. coli* adheres to intestinal epithelium and releases cytotoxins and enterotoxins in the extracellular lumen causing crypt orifice dilation, rounding and extrusion of enterocytes. Additionally, there are reports of cytoskeletal rearrangements in the host intestinal cells [134,135]. In ETEC, the production of heat liable and/or heat stable enterotoxins cause traveler’s diarrhea especially in elderly and children. Enteropathogenic *E. coli* causes similar pathology by producing heat labile and heat stable enterotoxins. However, the toxins of EPEC are distinct from those produced by ETEC. In addition, EPEC associated diarrhea involves the enteroadherence and production of toxin like Shiga toxin [136].

There are several reports on the efficacy of plant extracts and plant derived antimicrobials in inhibiting *E. coli* toxin production and reducing toxin mediated cellular damage on host cells. Natural extracts from *Galla chinensis* and *Berberis aristata* have been used in traditional Chinese medicine for the treatment of diarrhea [88,89]. Chen and coworkers [90] investigated the effect of *Galla chinensis* and gallic acid, one of the major components of *Galla chinensis* extract, on ETEC toxin binding to GM1 ganglioside receptor of humans. These researchers observed that *Galla chinensis* extract and gallic acid were effective in blocking the binding of heat labile enterotoxin to GM1 receptor *in vitro.* Follow up mouse gut assay revealed that *Galla chinensis* extract was effective in reducing toxin-mediated diarrhea indicating its efficacy in reducing toxin binding and toxin mediated cellular pathology. In a similar study, Chen and coworkers [91] observed that zingerone (vanillylacetone), one of the active components of ginger, was effective in preventing binding of heat labile toxin to GM1 and prevented accumulation of diarrheal fluid in the closed ileal loops of mice. In a recent study, Dubreuil and coworkers [92] observed a significant reduction (70%) in ETEC toxin-mediated diarrhea with plant alkaloid berberine (benzyltetrahydroxyquinoline) in a rabbit ileal loop model. These researchers also found an inhibition in the secretory response of the heat-stable toxin in infant mouse [92]. A similar anti-toxic activity with other plant derived compounds namely, oleanolic acid, ursolic acid, and betulinic acid [93], λ-carragenan [94], essential oil from *Cymbopogon martini* and *C. winterianus* [95], *Psidium guajava* [96], thiols and disulfide compounds [97] has been reported.

In addition to inhibiting toxin binding with receptor, plant compounds have also been found to modulate toxin production in pathogenic *E. coli.* As mentioned earlier, the pathogenesis of EHEC is primarily associated with the production of verotoxins. Sakagami and coworkers [98], investigated the effect of extracts from four plant species namely, *Limonium californicum* (Boiss.) A. Heller, *Cupressus lustianica* Miller, *Salvia urica* Epling and *Jusiaea peruviana* L., on verotoxin production *in vitro*. These researchers observed that plant extracts at concentrations below MIC reduced verotoxin production *in vitro.* Similarly, Takemasa and coworkers [99] found a concentration dependent reduction in extracellular and intracellular verotoxins by eugenol. Likewise, a decrease in toxin production has been reported following treatment with plant extracts such as catechin [100], epigallocatechin [101], cinnamon bark oil, cinnamaldehyde, and eugenol [102]. In addition, plant extracts, including green tea extracts, tea catechins, and green tea polyphenol were found to modulate other virulence attributes in EHEC such as biofilm formation, swarming motility and quorum sensing [103]. Since the concentrations of plant compounds used in above studies were below MIC, the effects observed were not due to reduction in cell number, but probably due to modulation of gene transcription in cells. In a recent study, Doughari and colleagues [104] found that *Curtisia dentata* extract reduced the transcription of *stx1* and *stx2* genes in EHEC. A similar reduction in the expression of *stx1* and *stx2* genes was observed with sub-inhibitory concentrations of other plant compounds such as *trans*-cinnamaldehyde, eugenol, carvacrol, thymol, and beta-resorcylic acid [105]. These researchers also reported that the aforementioned plant compounds reduced the expression of globotriaosylceramide (Gb3) receptor on human lymphocytes in addition to their effect on toxin genes. A similar host protective effect was observed with grape seed and pomace extracts using a novel cell-based assay *in vitro* [106].

In addition to reducing toxin production and toxin-receptor binding, receptor-mimicking compounds are being used as a novel strategy for combating toxins and other pathological agents. A handful of studies have investigated the potential of plant compounds as phantom receptors to ameliorate the effect of toxins. Kulkarni and coworkers [107] investigated the efficacy glycan encapsulated gold nanoparticles to selectively inhibit shiga toxins 1 and 2, and observed that the designed nanoparticles were effective in reducing toxin mediated pathology by mimicking the glycans in lipid rafts of the glycolipids receptors. Similar results were obtained with shiga toxin targeting synthetic ligands [108] and trisaccharide carrying carbosilane dendrimers [109] that mimics *stx* receptor. These studies indicate that plant compounds act not only by decreasing toxin-receptor binding but also through modulation of critical toxin genes and host receptor expression, thereby leading to reduced toxin-mediated pathology.

### 4.3. Efficacy of Plant-Derived Antimicrobials in Reducing Fungal Toxins

The kingdom Fungi consists of eukaryotic microorganisms such as yeasts, molds and the more familiar mushrooms. Fungi are capable of producing a large number of toxins with diverse modes of action that can cause disease and death in humans and animals. The major fungal toxins include mycotoxins (produced by molds) and toxins produced by mushrooms. Mycotoxins exert their toxic effects when humans or animals inadvertently consume, or come in contact with a substrate that contains the toxin-producing fungus or the mycotoxin. Mushroom poisoning on the other hand usually occurs after deliberately ingesting the basidiocarp [137]. Since not many studies have investigated the effect of plant compounds on mushroom poisons, this review focused primarily on the effect of plant compounds on mycotoxins production in this section.

Unlike bacterial toxins, mycotoxins are secondary metabolites produced by molds, and are not essential for growth of the producing fungus. Mycotoxins are low molecular weight, differentiation products with diverse chemical structures biosynthesized by complex cellular pathways [138,139]. Mycotoxins contaminate food crops or animal feed either before or after harvest and their concentration increases during postharvest storage. Mycotoxins reach consumers through the consumption of contaminated plant materials or via contaminated animal products such as meat, milk and egg. The normal industrial processing cannot remove mycotoxins from food products since they are heat-resistant within the range of conventional food-processing temperatures (80–121 °C). This is attributed primarily to their stable chemical structure [140,141]. The economic losses associated with mycotoxin contamination of crops are estimated to range between $500 million and $1.5 billion a year in the United States [142,143].

Mycotoxins commonly found in human food products and animal feed include *ochratoxin A*, *patulin*, *zearalenone*, *trichothecenes*, *fumonisins*, and *aflatoxins*. These mycotoxins are hazardous to animal and human health due to their carcinogenic, mutagenic, and teratogenic properties [144,145]. Current methods employed to control mycotoxin contamination use synthetic fungicides. Because of the non-biodegradable nature of these synthetic chemicals, they accumulate in soil, plants, and water, eventually leading to food contamination. This poses significant public health concerns. In addition, acute toxicity and hormonal imbalance in animals and humans have been observed [141,146]. In order to reduce the utilization of synthetic chemical fungicides in foods, several alternative treatments have been studied. Among the alternative approaches, the use of plant-derived antimicrobials has gained significant attention due to their natural, eco-friendly, and bio-degradable characteristics [141,145,147]. Research highlighting the potential use of plant compounds in reducing mycotoxins is described below.

*Ochratoxin A* produced by *Aspergillus ochraceus* and *Penicillium verrucosum* contaminates foods and feedstuffs of cereal origins [110,148], and causes renal toxicity, nephropathy, immunosuppression and even cancer in several animal species [149,150]. Basilico and coworkers [110] tested the effect of plant-derived oils namely oregano, mint, basil, sage, and coriander on *A. ochraceus* growth and *ochratoxin A* production *in vitro.* Results revealed that oregano and mint at 1000 ppm completely inhibited the fungal growth and ochratoxin A production up to 21 days, whereas basil was only effective up to seven days.

*Penicillium expansum* or blue mold is a serious postharvest pathogen that causes decay on apple, pear, peach, and grape fruits. This fungus produces toxin patulin in spoiled or processed fruits [111]. *Patulin* has been reported to be genotoxic, embryotoxic, and immunosuppressive in animals and humans [151,152,153,154]. Ikeura and coworkers [111] investigated the effect of various plant-extracts on *P. expansum* growth *in vitro.* Extracts from garlic, thyme, and lavender oils were found to effectively inhibit the formation of *P. expansum* spores. Follow up studies on apples revealed that the aforementioned plant extracts reduced fungal growth on the fruit. Among the tested plant extracts, garlic extract was most effective in inhibiting *P. expansum* growth on apples. In another study, Mossini and coworkers [112] investigated the effect of *Azadirachta indica* extracts on *P. expansum* growth and patulin production *in vitro*, and found the extracts were effective to inhibiting growth of *P. expansum* as well as *patulin* production.

Several *Fusarium* species produce toxins of significant concern to livestock and poultry producers. For example, zearalenone also known as RAL is primarily produced by *Fusarium graminearum.* It contaminates cereal crops such as wheat, barley, rice sorghum and maize. Zearalenone binds to estrogen receptors and results in hormonal changes that lead to abortion, infertility and other breeding problems in animals, especially swine [155,156,157]. Deoxynivalenol, a type B trichothecene, is produced by *Fusarium* that contaminates cereal grains. Deoxynivalenol binds to the ribosome and activates critical cellular kinases involved in signal transduction related to proliferation, differentiation, and apoptosis in human cells, and eventually disrupts normal cell function by inhibiting protein synthesis [158], thereby causing immunosuppression and kidney damage [159]. *Fumonisins B* is another important class of mycotoxins synthesized by *F. proliferatum* [160]. When ingested, they can lead to liver cancer and pulmonary edema in animals and esophageal cancer in humans [161]. They are commonly found in maize [162] and other plant tissues, including asparagus [163], pineapple [164] and garlic [165].

Mari and coworkers [113] investigated the effect of *cinnamon*, *clove*, *oregano*, *palmarosa*, and *lemongrass* oils on zearalenone and deoxynivalenol production by *F. graminearum* in maize. *Lemongrass*, and *palmarosa* significantly reduced zearalenone production, whereas clove was found to be the most effective treatment that inhibited the production of both zearalenone and deoxynivalenol. In another study, Stepien and coworkers [114] observed that extracts from various plant sources such as maize, garlic, and pea modulated the growth and fumonisin synthesis in *F. proliferatum* strains *in vitro.* Pea extract was found to be the most efficient inhibitor of *fumonisin* synthesis in the mold.

*Aflatoxins* produced by *Aspergillus flavus* and *Aspergillus parasiticus* are one of the most potent mycotoxins because of their high toxicity, carcinogenicity as well as their biotransformation ability in the animal and human body. They primarily contaminate commonly used feed ingredients such as peanut, corns, and cottonseeds resulting in severe negative health effects in both animals and humans [166]. In animals, consumption of aflatoxin contaminated feed causes poor feed utilization, decreased body weight gain, reduced egg production and increased mortality [167,168,169]. Moreover, *aflatoxin* residues can be found in animal products such as milk, meat and egg [170], and are metabolized and transformed to *aflatoxin M1* and *M2*. *Aflatoxin B1* is listed as group 1 human carcinogen by the International Agency for Research on Cancer, and mainly causes liver cancer [171,172]. *Aflatoxins* are also known to interact with hepatitis B virus leading to human hepatocellular carcinoma [173]. According to the current U.S FDA regulations, the maximum permissible limit of *aflatoxins* in human and young animal food is 20 ppb, whereas for adult livestock it is 100 ppb [174].

Significant research is being conducted to investigate the potential of natural antimicrobials in inhibiting the growth and aflatoxin production by *Aspergillus* species in food products and animal feed. Clove and cinnamon oils at 100 ppm were found to inhibit *A. flavus* growth and *aflatoxin* production *in vitro* [115]. In the same study, the researchers demonstrated that cinnamon oil at 1000 ppm exhibited maximum inhibitory action and reduced *aflatoxin* production by 78% on maize. Similarly, *Zataria multiflora* was found to exert significant inhibitory effect on both *A. flavus* growth and *aflatoxin* production in cheese [116]. Follow up mass spectrometric analysis revealed that the major component (71%) of *Zataria multiflora* oil was carvacrol. The antitoxin effect of carvacrol on *A. parasiticus* was investigated by Razzaghi-Abyaneh and coworkers [117]. These researchers reported that carvacrol effectively suppressed the growth as well as the synthesis of *aflatoxin B1* and *G1* by *A. parasiticus in vitro*. In another study, carvacrol and trans-cinnamaldehyde were found to significantly reduce growth and aflatoxin production in *A. flavus* and *A. parasiticus in vitro.* In addition, these compounds also reduced the toxin production in poultry feed [51]. The two plant compounds also down-regulated the expression of *aflatoxins* synthesis genes (*aflC*, *nor1*, *norA*, and *ver1*) in the molds. Follow up *in vivo* studies showed that in-feed supplementation of carvacrol and *trans*-cinnamaldehyde was effective in reducing aflatoxicosis in broiler chickens that were fed with mold-contaminated feed. In addition, histological analysis of liver from chickens fed with plant compounds revealed significantly lesser hepatocellular degeneration, inflammation and necrosis as compared to control birds [175]. Similar inhibitory effect on *aflatoxins* production was obtained with other plant-derived antimicrobials, namely *Thymus eriocalyx* [176], *Thymus vulgaris* [177], *thyme*, *anise*, *cinnamon*, and *spearmint* [178]. These results suggest that plant compounds could be used as feed/food additives to control *aflatoxins* contamination in food products or animal feed.

## 5. Conclusions and Future Directions

Research efforts in the past few decades have significantly improved our understanding of microbial virulence and toxin production. Disruption of toxin production and toxin-mediated pathology in hosts is widely regarded as a potential anti-virulence strategy since this approach, being non-microbicidal, would circumvent the selective pressure for resistance development. The aforementioned information collectively suggests that plant-derived antimicrobials could be used to control toxin production in microorganisms, and toxin mediated pathology in humans and animals. With advancements in nanotechnology and pharmaceutics, future research should focus on improving the drug delivery technology to improve the efficacy of plant compounds at target sites. Moreover, in-depth studies are required to properly characterize the therapeutic/chemical effects of plant compounds in the host for developing reliable and effective drugs. In addition, studies are required to minimize the changes in organoleptic properties of food products when supplemented with plant-derived compounds to further improve their applications, especially in the food industry.
